# Reduced age-associated brain changes in expert meditators: a multimodal neuroimaging pilot study

**DOI:** 10.1038/s41598-017-07764-x

**Published:** 2017-08-31

**Authors:** Gaël Chételat, Florence Mézenge, Clémence Tomadesso, Brigitte Landeau, Eider Arenaza-Urquijo, Géraldine Rauchs, Claire André, Robin de Flores, Stéphanie Egret, Julie Gonneaud, Géraldine Poisnel, Anne Chocat, Anne Quillard, Béatrice Desgranges, Jean-Gérard Bloch, Matthieu Ricard, Antoine Lutz

**Affiliations:** 1 Inserm, Inserm UMR-S U1237, Université de Caen-Normandie, GIP Cyceron, Caen, France; 2 Normandie Univ, UNICAEN, EPHE, INSERM, U1077, CHU de Caen, Neuropsychologie et Imagerie de la Mémoire Humaine, 14000 Caen, France; 3 Rheumatology, 67000 Strasbourg, France; 4 Shechen Monastery, P.O. Box 136 Kathmandu, Nepal; 5 Lyon Neuroscience Research Center INSERM U1028, CNRS UMR5292, Lyon 1 University, Lyon, France

## Abstract

Aging is associated with progressive cerebral volume and glucose metabolism decreases. Conditions such as stress and sleep difficulties exacerbate these changes and are risk factors for Alzheimer’s disease. Meditation practice, aiming towards stress reduction and emotion regulation, can downregulate these adverse factors. In this pilot study, we explored the possibility that lifelong meditation practice might reduce age-related brain changes by comparing structural MRI and FDG-PET data in 6 elderly expert meditators versus 67 elderly controls. We found increased gray matter volume and/or FDG metabolism in elderly expert meditators compared to controls in the bilateral ventromedial prefrontal and anterior cingulate cortex, insula, temporo-parietal junction, and posterior cingulate cortex /precuneus. Most of these regions were also those exhibiting the strongest effects of age when assessed in a cohort of 186 controls aged 20 to 87 years. Moreover, complementary analyses showed that these changes were still observed when adjusting for lifestyle factors or using a smaller group of controls matched for education. Pending replication in a larger cohort of elderly expert meditators and longitudinal studies, these findings suggest that meditation practice could reduce age-associated structural and functional brain changes.

## Introduction

Aging is associated with a number of changes in the brain that, collectively, contribute to the decline in cognitive function observed in older adults. Neuroimaging studies have allowed us to track age-related macroscopic, structural, functional and molecular brain changes. They have shown substantial decreases with age in cerebral volume and glucose metabolism^1, 2^. These changes are not homogeneous throughout the brain as they predominate in the frontal cortex and are also often reported in the anterior cingulate cortex, insula, sensorimotor, and perisylvian regions^1–3^. Other parietal and temporal brain regions, including the hippocampus, seem to be involved as well, yet findings are less consistent across studies. Age-related decreases in brain structure and function are known to be associated with decline in cognitive performance, especially in executive functions and episodic memory^2, 4^. Age is also associated with a significant increase in β-amyloid (Aβ) deposition, as measured with positron emission tomography (TEP) using different amyloid radiotracers^5, 6^. Decreased gray matter (GM) brain volume (especially in the hippocampus and temporal neocortex), and glucose metabolism (in the posterior cingulate cortex and temporo-parietal region), and the presence of Aβ deposition, are known to be associated with increased risk for dementia, and particularly for Alzheimer’s disease (AD).

It is increasingly acknowledged that several lifestyle factors modulate brain aging and the development of dementia; around a third of AD cases may be attributable to potentially modifiable risk factors^7^. These findings are of considerable interest as they suggest that a modification in these lifestyle factors might allow for the promotion of healthy brain aging, prevent or delay AD, and reduce AD risks. Thus, higher levels of cognitive and physical activity have been shown to be associated with higher brain volume and metabolism, lower cerebral Aβ deposition, and lower risk for cognitive decline and dementia in cognitively normal elderly^8–11^. Similarly, psycho-affective factors such as depression, stress and anxiety – and sleep difficulties often associated with these conditions – have also been identified as risk factors for AD, having a negative impact on brain structure and function, and reducing the mental health and well-being of the aging population. Moreover, stress has a detrimental effect on hippocampal integrity^12^, sleep disorders foster AD-related pathological processes^13^, and depressive symptoms in older persons are associated with an increase in dementia risk^14^.

Mental training for stress reduction and emotion regulation through meditation practice has the potential to downregulate various adverse factors^15, 16^, and thus could positively affect neurological conditions, and promote mental health and wellbeing in the aging population^17^. While meditation research is still in its infancy especially in elderly populations, there is emerging evidence that meditation practice improves cognition, mainly attention, but also memory, which are the most sensitive to aging and AD^18^. It has also been shown to reduce stress, anxiety, depression, insomnia, feelings of loneliness and social exclusion^17, 19^, and cardiovascular risk factors in older adults^20^. A previous study showed reduced age-effects on the volume of the putamen in meditators compared to controls aged 25 to 45 years^21^. Moreover, the effects of meditation on brain structure and function have consistently been reported in young and middle-aged adults, especially in frontal and limbic structures, as well as the insula^22, 23^.Interestingly, these structures are known to be particularly sensitive to aging and AD. Yet, only one research team investigated this question in the context of an elderly population, assessing brain volume in expert meditators and controls aged between 24 and 77 years. They showed that the regression line between GM volume and age was steeper in controls than in meditators, particularly in frontal and temporal brain regions^24, 25^. No study to date has explored changes in glucose metabolism associated with long-term meditation practice in young or elderly participants.

The present pilot study aimed at providing an overall picture of structural and functional brain changes in elderly expert meditators by measuring cerebral volume and glucose metabolism with MRI and FDG-PET scans. As a secondary objective, we investigated whether the brain regions exhibiting meditation-expertise-related effects were sensitive to aging and whether the elderly expert meditators differed from matched controls in this aging process. Additionally, complementary analyses were conducted to assess changes in cognition, lifestyle, and self-perceived sleep quality in the expert meditators, as well as to explore the hippocampal substructures in more detail. 192 participants were included in this study, including six expert meditators aged 60–70 years, and 186 controls aged 20–87 years. The whole control group was used to map age-related brain changes, while a group of 67 elderly controls (those aged 55–75) was used for direct comparison to the elderly expert meditators.

## Results

The elderly expert meditators did not differ in age, sex ratio, nor MMSE scores in comparison to the elderly control group, yet showed significantly higher years of education (Table 1). Among the participants who had a Florbetapir-PET scan, 10/61 were classified as Aβ-positive (16%), while all 4 elderly expert meditators were classified as Aβ-negative (Supplementary Figure S1). Due to these differences in years of education and rate of Aβ-positive individuals, all neuroimaging comparisons were also performed using a subgroup of Aβ-negative elderly controls matched for education (see complementary analyses below).

**Table 1:** Demographics

	Healthy controls			Elderly expert meditators
	Whole sample	Elderly controls	Education-matched Aβneg elderly subgroup	
Sample size	186	67	31	6
Age mean ± SD (range)	49.1 ± 18.7 (20–85)	64.8 ± 6.4 (55–75)	64.5 ± 6.9 (55–75)	64.8 ± 3.2 (61–70)
Education mean ± SD (range)	13.1 ± 3.2 (7–20)	12.1 ± 3.7* (7–20)	15.1 ± 2.6 (12–20)	16.2 ± 2.7 (12–20)
N females/males	97/89	38/29	20/11	3/3
MMSE mean ± SD (range)	—	29.1 ± 1.0 (26–30)	29.2 ± 1.0 (26–30)	29.5 ± 0.8 (28–30)

Mann-Whitney U-tests and chi2 statistics were performed to compare elderly expert meditators to the elderly control group (*Significant difference from the elderly expert meditators, p < 0.05). MMSE: mini mental state examination. SD: standard deviation.

### Main analyses

**Figure 1:**
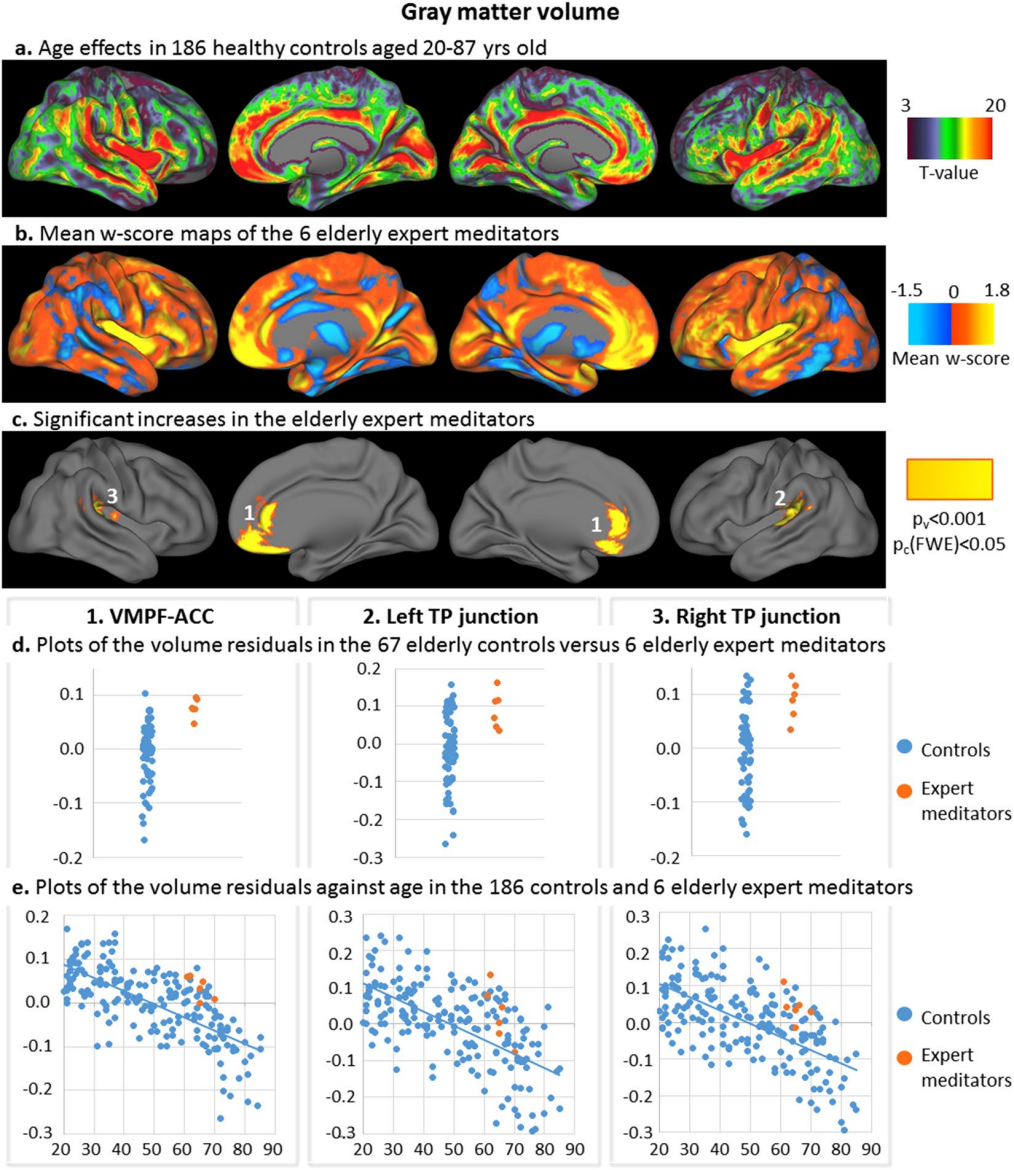
Results of the analyses on gray matter volume. T-values of the voxelwise regression analysis between the z-score maps of the 186 controls (corrected for education) and age were superimposed on brain surface views (**a**). Mean w-score values of the expert meditators corrected for age and education and using the elderly control group as the reference **(b**), and clusters of interest showing significantly higher values in the expert meditators (voxel-level p < 0.001 and FWE-corrected cluster-level p < 0.05) (**c**) were superimposed on brain surface views. Volume residuals in the 3 clusters of interest were plotted in the elderly expert meditators (orange) and in the controls (blue) of the elderly control group (**d**; residuals are corrected for age and education); of the entire control group against age (**e**; residuals are corrected for education). **VMPF-ACC**: ventromedial prefrontal and anterior cingulate cortex; **TP**: temporo-parietal.

**Figure 2:**
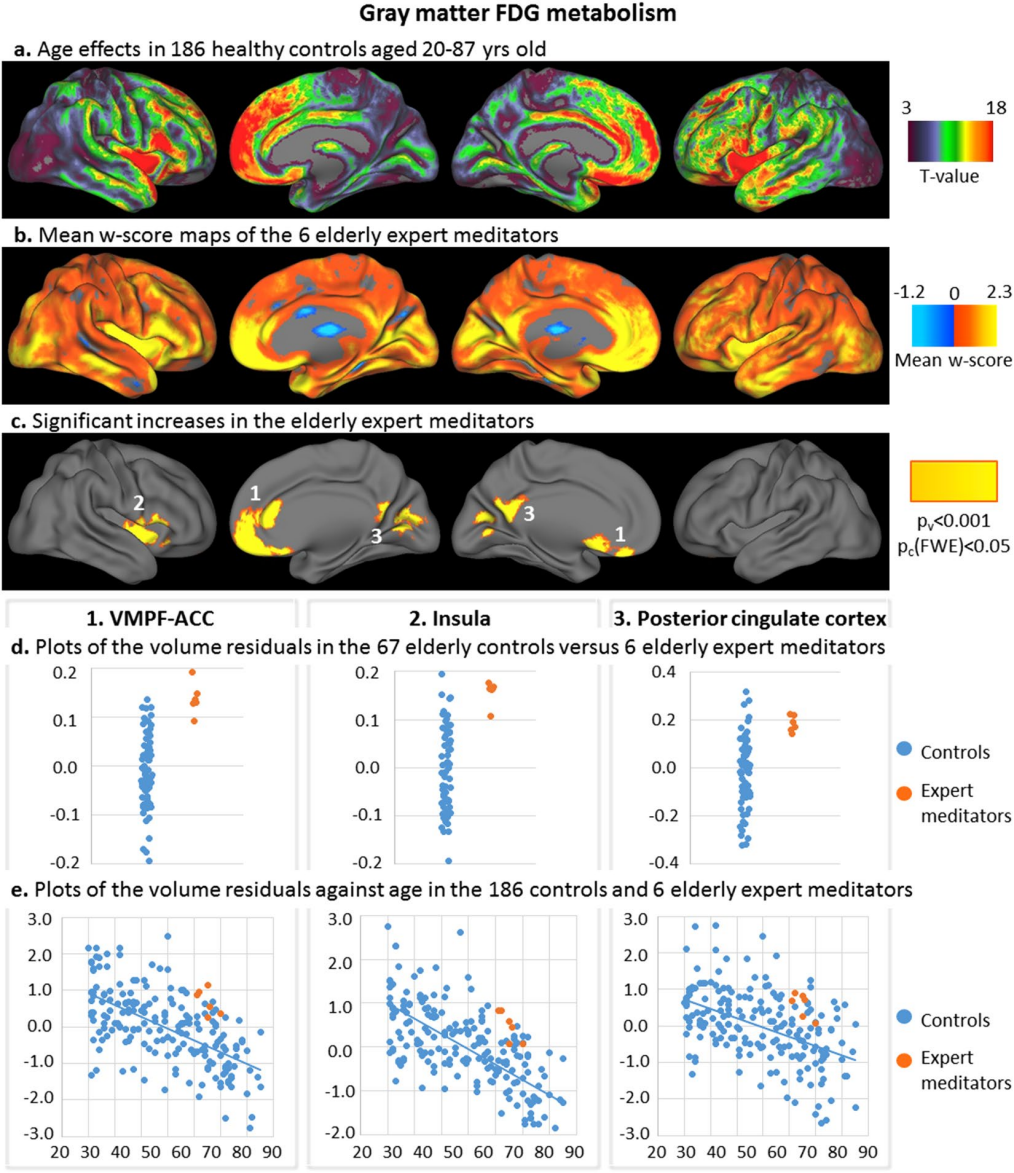
Results of the analyses on gray matter glucose metabolism. T-values of the voxelwise regression analysis between the z-score maps of the 186 controls (corrected for education) and age were superimposed on brain surface views (**a**). Mean w-score values of the expert meditators corrected for age and education and using the elderly control group as the reference (**b**), and clusters of interest showing significantly higher values in the expert meditators (voxel-level p < 0.001 and FWE-corrected cluster-level p < 0.05) (**c**) were superimposed on brain surface views. Glucose metabolism residuals in the 3 clusters of interest were plotted in the elderly expert meditators (orange) and in the controls (blue) of the elderly control group (**d**; residuals are corrected for age and education); of the entire control group against age (**e**; residuals are corrected for education). **VMPF-ACC**: ventromedial prefrontal and anterior cingulate cortex; **TP**: temporo-parietal.

Structural MRI and FDG-PET data of elderly expert meditators were transformed in w-score maps using the elderly control group as the reference and adjusting for age and education. The mean w-score maps of the elderly expert meditators showed that w-score values were mainly positive for GM volume (Fig. 1B), and almost only positive for FDG metabolism (Fig. 2B), indicating that the elderly expert meditators tended to have higher values compared to elderly controls. The highest mean w-scores were found in the insula, medial frontal and temporal cortical areas for both GM volume and FDG metabolism. More specifically, the z-scores were significantly higher than zero, the reference for the elderly controls, in three clusters for GM volume (Fig. 1C) and in three clusters for FDG metabolism (Fig. 2C), referred to as the clusters of interest in what follows. Thus, GM volume was significantly increased in the elderly expert meditators in the ventromedial prefrontal and anterior cingulate cortex bilaterally (MRI-cluster 1), and in the left (MRI-cluster 2) and right (MRI-cluster 3) medial temporo-parietal junction encompassing the transverse temporal gyrus, planum temporale, parietal operculum, and posterior extremity of the insula (long gyrus). FDG metabolism was significantly increased in the elderly expert meditators in the ventromedial prefrontal and anterior cingulate cortex bilaterally (FDG-cluster 1), in the right insula (mainly the anterior section i.e. the short gyrus), also encompassing the pars opercularis of the inferior frontal gyrus (FDG-cluster 2), and in the posterior cingulate cortex extending to the precuneus and cuneus (FDG-cluster 3). The plots of the individual values (Figs 1D and 2D) show that, for all regions, the values of the elderly expert meditators fall within the upper limit of those of the controls (or were still above the highest values of the controls for FDG-cluster 1). The reverse contrasts, that is the lower GM volume or FDG metabolism in the elderly expert meditators, did not reveal any significant results.

In the 186 controls aged 20–87 years, age-related decreases in cortical GM volume and FDG metabolism were found predominantly in the bilateral insula, medial frontal, cingulate, and temporal neocortical areas (Figs 1A and 2A). Decreases in GM volume also included the cuneus and temporo-parietal junction, while age-related decreases in FDG metabolism predominated in anterior brain regions. Increased GM volume or FDG metabolism in elderly expert meditators concerned regions that were primarily affected by age, with the exception of the posterior cingulate cortex, which showed only moderate age-related FDG metabolism decreases (FDG-cluster 3). Regression plots showing GM volume and FDG metabolism as a function of age in the clusters of interest (Figs 1E and 2E) show that elderly expert meditators had higher volume and metabolism in the clusters of interest than expected for their age; all individual values of elderly expert meditators were above the slope of the regression with age in the controls. Interestingly, the voxels showing the strongest age-effects (25% highest t-values) on GM volume and FDG metabolism overlapped 75% and 60% respectively of the MRI and FDG clusters showing an effect of meditation, i.e. the clusters of interest.

### Complementary analyses

Results of complementary analyses on cognition and lifestyle are reported in the Supplementary Table S1. There was no significant difference in the cognitive performance between the elderly expert meditators and the elderly control group, but a marginal effect was observed for episodic memory, with a trend for higher performances in the elderly expert meditators. With regard to lifestyle, a significant difference was found for the leisure activity score before 30 years, and a trend was observed for all other measures in non-parametric statistics. However, in the ANOVA correcting for years of education, only the trend effect for diet remained as the expert meditators tended to have a greater adherence to the Mediterranean diet than did controls. As for sleep measures, a difference was found only for duration of awakenings, with expert meditators showing shorter duration than controls, the difference being significant in the non-parametric test and marginal in the ANOVA correcting for education. When assessing hippocampal subfield volumetry, the elderly expert meditators tended to have larger CA1 subfield volumes compared to controls (Supplementary Table S1). The projection on the 3D surface view of the hippocampus showed that meditation-expertise-related effects on the hippocampus were weak and mainly concerned the CA1 subfield (predominantly in the left hemisphere), and to a lesser degree the subiculum (on left and right inferior views; Supplementary Figure S2).

The differences in GM volumes and FDG metabolism in the clusters of interest between the elderly expert meditators and the elderly controls remained essentially the same when performing ANOVAs including education and all lifestyle measures as covariates (Supplementary Table S2), or when using a subgroup of 31 Aβ-negative elderly controls matched for education (Table 2).

**Table 2:** Between-group differences in gray matter volume and FDG metabolism in the clusters of interests (shown in Figs 1C and 2C) when comparing the 6 elderly expert meditators versus the 67 elderly controls or the 31 Aβ-negative elderly controls matched for education (p-values; Mann-Whitney U-tests).

	67 elderly controls	31 education-matched Aβneg elderly controls
MRI-cluster 1: VMPC-ACC	0.0003	0.00008
MRI-cluster 2: L TP junction	0.008	0.01
MRI-cluster 3: R TP junction	0.002	0.003
FDG-cluster 1: VMPC-ACC	0.0001	0.0003
FDG-cluster 2: R Insula	0.0003	0.0007
FDG-cluster 3: PCC	0.001	0.0009

**VMPF:** ventromedial prefrontal cortex; **ACC**: anterior cingulate cortex; **TP**: temporo-parietal; **PCC:** posterior cingulate cortex; **L:** left; **R:** right.

## Discussion

The main aim of this pilot study was to explore differences in GM volume and glucose metabolism in elderly expert meditators compared to elderly controls. We found higher volume and glucose metabolism in the anterior cingulate and ventro-medial prefrontal cortex, insula, temporo-parietal junction (for volume) and posterior cingulate cortex (for metabolism) in the elderly expert meditators. The findings need to be interpreted with caution given the small sample size of the elderly expert meditators. Yet, this study is the first to measure FDG-PET changes in meditators and it suggests that brain glucose metabolism is a sensitive measure for detecting changes associated with meditation practices. Meditation-expertise related metabolism changes included brain regions which spatially overlapped with the meditation-expertise related structural changes observed in our study (e.g. the anterior cingulate and ventro-medial prefrontal cortex), which were consistent with previous research on young and middle-aged meditators (e.g. the insula, see below). These effects were more marked than those found with structural MRI scans.

Our findings of increased brain volume and metabolism in elderly expert meditators and the topography of these findings are consistent with previous literature. Luders *et al*.^25^ showed markedly less age-related brain atrophy in expert meditators aged between 24 and 77 years compared to controls in extended brain areas including those highlighted in this study. In elderly participants, expert meditators were thus expected to show larger brain volume than controls in (at least some of) these brain regions, and our results confirmed this expectation. Moreover, studies assessing brain structure in young and middle-age meditation practitioners have reported higher volume compared to non-meditators in the insula, frontal, anterior cingulate, inferior temporal cortex, hippocampus and putamen, as well as the temporo-parietal junction and posterior cingulate cortex - precuneus^16^. In a meta-analysis from 21 structural neuroimaging studies examining nearly 300 meditation practitioners, changes in the insula and anterior cingulate cortex were found to be among the most replicated findings in these studies^22^. The anterior insula and anterior cingulate cortex are highly interconnected and together form the salience network, an intrinsic large-scale network involved in interoceptive awareness, i.e. self-awareness of one’s own body, respiration, heart rate, pain perception and emotional state^26, 27^. The insula is also involved in emotional and empathic processing, high-level cognitive control, and attentional processes^28^; the anterior cingulate is particularly associated with self-regulation of attention and emotion^22, 26, 29^. Meditation-related changes of activity in these regions were also consistently reported in fMRI studies^16, 22^. Thus, increased activity during mindfulness and/or compassion meditation in all or part of the salience network, anterior cingulate cortex and/or insula, was notably found to be associated with breath awareness^30^, heart rate^31^, pain perception^32^, emotional processing and empathy^33, 34^, and awareness of mind-wandering^35^. Our finding of increased anterior cingulate and insula volume/metabolism in elderly expert meditators is thus in line with the expected effects of meditation practice on brain structure and function based on these previous experiments.

The ventromedial prefrontal cortex is also consistently reported in structural neuroimaging studies on meditation practitioners, and is thought to underlie self-monitoring, affective theory of mind, and emotion regulation processes^16, 22, 36, 37^. Although less frequently involved, the temporo-parietal junction and posterior cingulate cortex – precuneus were also found to show increased volume in young and middle-aged meditators compared to controls^38, 39^. Altogether, these regions are parts of the default mode network (DMN) thought to be involved in self-referential processing, theory of mind, and mind wandering^40^. fMRI studies in meditators have shown decreased activity within the DMN, which has been interpreted as reflecting diminished self-referential processing and mind wandering^41^. By contrast, increased connectivity was found in meditators between DMN areas and anterior brain regions such as the anterior cingulate cortex, which was interpreted as reflecting increased cognitive control over the function of the DMN. Interestingly, lifelong elevated neuronal and metabolic activity within the DMN is thought to increase DMN hubs’ vulnerability to aging and AD pathological processes^42^. It is thus possible that DMN structures are relatively preserved in elderly expert meditators because they have endured lifelong reduced DMN activity compared to controls. Higher volume and metabolism in DMN structures in elderly expert meditators might thus reflect both a direct effect of meditation practice on brain structures involved in self-monitoring, emotion regulation, and cognitive control, and a protective effect of DMN activity regulation, resulting in better maintenance or preservation of these structures. Also, several structures of the DMN, such as the hippocampus, posterior cingulate and ventromedial prefrontal cortex, are known to be involved in episodic memory processes^42, 43^; the increased volume and/or FDG metabolism found in the elderly expert meditators might underlie the tendency for higher memory performances in our sample.

Regarding the hippocampus, morphometric differences have frequently been reported in meditators in this structure^22^. For instance, meditation practice was associated with increased left hippocampus volume in cognitively normal young and middle aged adults^38^, with trends for reduced bilateral hippocampal atrophy in patients with mild cognitive impairment^44^. Moreover, lighter age effects^24^ and larger volume^45^ were found in the subiculum of meditators aged 24 to 77 years compared to controls. In the present study, we found only marginal effects in the hippocampus of elderly expert meditators, with higher volume observed mainly in the CA1 subfield and part of the subiculum using a lenient statistical threshold. Our findings are thus partly consistent– although the volume of the CA1 subfield *per se* has not been assessed in previous research. The weakness of the effects in the present study might be due to the limited sample size of the meditator group and/or indicates that long-term meditation effects are less marked in the hippocampus, at least in elderly meditators, compared to those found in other brain structures. It is interesting to note that, while age effects are known to predominate in the subiculum, the CA1 subfield is the most sensitive to AD, especially in early prodromal stages of the disease^46, 47^.

Importantly, all the brain regions where elderly expert meditators showed higher volume and higher FDG metabolism were specifically those that are particularly sensitive to aging and/or AD. Thus, as highlighted in the Figs 1A and 2A, age effects in a large group of 186 healthy controls were found to be maximal in the insula, medial prefrontal,and anterior cingulate cortex, consistent with previous findings^1–3^. On the other hand, earlier AD-related structural/ glucose metabolism changes are known to concern the hippocampus or posterior cingulate cortex and temporo-parietal junction respectively^48^. This is particularly interesting in a perspective of brain reserve, maintenance and prevention, as the effects in expert meditators specifically concerned brain areas sensitive to aging and AD^9, 49^. It suggests that long-term meditation might help preserve the brain from the progressive deleterious effects of aging on brain volume and glucose metabolism, which in turn confers a brain reserve associated with a reduced risk of developing AD and/or a delay in the onset of the disease. Interestingly, previous studies in aging (or populations at-risk for AD) have shown increased volume and/or metabolism especially in the anterior cingulate cortex, but also in the medial prefrontal cortex, insula, and hippocampus in elderly with higher years of education, higher cognitive activity, or maintained cognitive functions^10, 50, 51^.

Our complementary analyses showed that elderly expert meditators also tended to have a more active lifestyle, both early on, including higher education, and later in life, a higher adherence to a Mediterranean diet, and better sleep quality (i.e., shorter duration of awakenings during the night). Some of these differences reflect initial differences in the samples, especially for early-life measures such as education and leisure activities.While we could not fully account for possible cohort effects, (see limitation paragraph below), adjusting for years of education and lifestyle factors and using a subsample of Aβ-negative elderly controls matched for education only poorly influenced our findings, suggesting that they were not merely reflections of these differences. Beyond neuroimaging, part of the between-group differences highlighted in our complementary analyses, especially those assessing later life / current periods, might reflect an impact of expert meditation on behavioural measures, including lifestyle, diet, and sleep. This result is again particularly interesting in the context of aging and Alzheimer’s disease, given the growing acknowledgment of the relevance of these factors (healthy diet, good sleep quality, more active lifestyle) for wellbeing and mental health, including decrease of AD risk in the aging population^7, 8, 10, 52^.

The present study has strengths and limitations. It is the first study to focus on elderly expert meditators, and the only study on meditation to include a measure of brain glucose metabolism. The large samples of controls who underwent the same examinations on the same scanners are also valuable as they allowed to refer to reliable normative data and to highlight aging effects across the entire adult lifespan. Despite the noise introduced by the heterogeneity in the styles of mindfulness or compassion meditation practiced in the various Buddhist traditions represented here (Zen, Dzogchen, Vipassana), we found brain regions commonly sensitive to meditation expertise. This finding suggests a generalizability of this effect beyond any particular tradition, but prevented us from identifying tradition-related effects. A larger sample of experts will be necessary to address this question. The main limitation of the present study is the small sample size of the elderly expert meditators (n = 6), which limited the statistical analyses we could conduct and their statistical power, and thus the interpretation of the findings. For instance, we could not assess reliable correlation analyses between neuroimaging and behavioural measures within the expert meditators, and the lack of significant differences in cognitive measures might be due to the limited sample size. Inversely, small sample size could also conduct to inflated or spurious effects^22, 53^. For instance, in studies assessing the impact of meditation on brain structures, the largest effect sizes were reported in studies with the smallest sample sizes, whereas more reasonable effect sizes were reported in well-powered, large sample size studies^22^. The cross-sectional nature of the study is also a limitation because of possible cohort effects. Only longitudinal studies would allow to test for a causal relationship between meditation and brain volume preservation in elderly populations. Overall, we think that this study provides encouraging findings to stimulate future research on meditation in the context of aging. Further studies with larger cohorts of elderly expert meditators and longitudinal studies assessing the effects of meditation on naïve elderly individuals are needed. Moreover, future research is needed to investigate the mechanisms underlying the effects of meditation, especially in the context of aging and AD.

## Methods

### Participants

A total of 192 participants were included in this study, including six expert meditators aged 60–70 years and 186 controls aged 20–87 years. The entire control group was used to map age-related brain changes, while a group of 67 elderly controls (those aged 55–75) was used for direct comparison to the elderly expert meditators (see Table 1 for demographics). All participants were involved in the *Imagerie Multimodale de la maladie d’Alzheimer à un stade Précoce* (IMAP+) Study (Caen, France), and part of the controls were included in previous publications from our laboratory^1, 8, 54, 55^. All participants were screened for the absence of history and clinical evidence for major neurological or psychiatric disorder and performed in the normal range on all tests of the screening neuropsychological battery, including the mini mental state examination (MMSE^56^).

Consistent with prior research on expertise, elderly expert meditators had at least 10,000 h of formal lifelong meditation practice. Meditators were trained in Buddhist meditation practices from various traditions including Zen Korean Buddhism (1 person), Tibetan Buddhism (4) and Vipassana (3). They all practiced some kind of mindfulness-related, loving-kindness, and compassion meditations. We deliberately chose to include a variety of Buddhist traditions, which practice these two families of practice, to be able to generalize our finding above and beyond any particular style of mindfulness or compassion meditation in these traditions. The underlined assumption is that these various sub-genres of mindfulness and compassion meditation could commonly impact aspects of attention, emotion and stress regulation, and psychoaffective factors known to impact on brain aging, and overall on mental health and well being in aging. Elderly expert meditators had between 15,000 to 30,000 hours of meditation practice over the last 10 to 40 years, about half of which was carried out at retreats.

The IMAP+ Study was approved by a regional ethics committee (Comité de Protection des Personnes Nord-Ouest III) and is registered with http://clinicaltrials.gov (number NCT01638949). All participants gave written informed consent to the study prior to participation. All experiments were performed in accordance with relevant guidelines and regulations.

### Imaging data acquisition

All participants underwent neuroimaging scans on the same MRI and PET scanners at the Cyceron Centre (Caen, France).

#### MRI data

MR scans were all acquired on a Philips Achieva (Eindhoven, The Netherlands) 3 T scanner. Subjects were equipped with earplugs and their heads were stabilized with foam pads to minimize head motion. T1-weighted structural images were obtained in all participants using a three-dimensional fast-field echo sequence (sagittal; repetition time = 0 ms; echo time = 4.6 ms; flip angle = 10°; 180 slices; slice thickness = 1 mm; field of view = 256 × 256 mm²; matrix = 256 × 256). Moreover, a high resolution proton density (PD) weighted sequence was acquired perpendicularly to the long axis of the hippocampus (TR = 3500 ms; TE = 19 ms; flip angle = 90°; 13 slices; slice thickness = 2 mm; inter-slices gap = 2 mm; in-plane resolution = 0.375 × 0.375 mm², acquisition time = 7.4 min), in the 6 elderly expert meditators and in 53 of the 67 older controls. This last scan was used for hippocampal subfield manual delineation.

#### PET data

All participants had a FDG-PET scan within 2 months of the 3D-T1 MRI. Sixty-one of the elderly control group and 4 of the expert meditators also had a Florbetapir-PET scan to measure Aβ deposition. Both FDG- and Florbetapir-PET scans were acquired on a Discovery RX-VCT-64 PET-CT device (GE Healthcare) with a resolution of 3.76 × 3.76 × 4.9 mm (field of view = 157 mm). Forty-seven planes were obtained with a voxel size of 2.7 × 2.7 × 3.27 mm. A transmission scan was performed for attenuation correction before the PET acquisition. For FDG–PET scans, participants fasted for at least 6 hours before scanning and were at rest in a quiet and dark room during the 30 minutes preceding tracer injection. Intravenous injection of approximately 180 MBq of FDG was carried out 50 minutes before a 10-minute acquisition. Regarding the Florbetapir-PET scan, intravenous injection of approximately 4 MBq/kg of Florbetapir was carried out 50 minutes before a 20-minute acquisition.

### Imaging data pre-processing

*T1-weighted MRI* were segmented, normalized, and modulated for nonlinear warping using the Segment function of the Statistical Parametric Mapping 12 (SPM12) software (Wellcome Trust Centre for Neuroimaging, London, UK, http://www.fil.ion.ucl.ac.uk/spm/software/spm12/). Resulting local GM volume maps corrected for brain size were finally smoothed with a Gaussian kernel of 8 × 8 × 8 (x, y, z).

*FDG and Florbetapir PET data* were first corrected for partial volume effects (PVE, PMOD Technologies) using a 2-tissue compartment model. Resultant images (GM compartment only) were coregistered onto their corresponding MRI, and normalized using the deformation parameters derived from the MRI. Images were then scaled using the cerebellar GM as a reference. Normalized and scaled FDG-PET data were smoothed with a Gaussian kernel of 10 × 10 × 10 (x, y, z). For Florbetapir-PET data, a global neocortical mean value was extracted from each individual using a predetermined mask, (including all regions but the cerebellum, hippocampus, amygdala and subcortical gray nuclei), and participants were classified as Aβ-positive or Aβ-negative based on Florbetapir-PET data acquired in a group of 41 healthy adults under 40 years old^54, 57^.

### High resolution PD-weighted hippocampal acquisition

Hippocampal subfields were manually segmented on the high resolution Proton Density weighted scan according to a protocol detailed in previous publications^46, 58^. Briefly, three hippocampal regions were delineated: (i) the subiculum; (ii) CornuAmmonis (CA) 1; and (iii) CA2, CA3, CA4, and the Dentate Gyrus (DG) pooled together in a unique region termed CA2/3/4/DG in what follows. Manual delineations were all performed by the same raters as in our previous publications showing high inter-rater reliability (ICC values between the two raters = 0.8 to 0.9). The volume of the whole hippocampus corresponded to the sum of the volumes of the three resulting hippocampal regions.

### Neuropsychological tests and lifestyle and sleep questionnaires

Neuropsychological measures and questionnaire scores were selected among detailed assessments included in the IMAP+ study and are fully described in our previous publications^59, 60^. Here we selected a few representative measures of cognition and self-reported assessment of lifestyle and sleep quality. We used one score for each of the following cognitive areas: verbal fluency (sum of category and letter fluency tasks), episodic memory (sum of the free recall of two 16-word lists from the ESR task^59^), short-term memory, and working memory (forward and backward digit span), processing speed and executive functions (Trail Making Test part A and inhibition score part B – part A, respectively). Moreover, we used scores derived from the Lifetime of experiences questionnaire (LEQ^61^) to assess complex mental activity early and later in life. Specifically, we derived two scores reflecting the frequency of participation in leisure activities before 30 and from 30 to 65 years, and a third score reflecting the highest level of occupation reached from 30–65 years. Adherence to Mediterranean diet was quantitatively assessed using the Mediterranean Adherence Screener (MEDAS, Schröder *et al*., 2011). Finally, sleep was assessed using a questionnaire derived from the Pittsburgh Sleep Quality Index (PSQI^62^), assessing sleep quality and disturbances during the last five years. This questionnaire, previously used by our research group^52^, allows to extract the following information: sleep quality, sleep latency and duration, as well as the number and duration of nocturnal awakenings.

### Statistical analyses

#### Main analyses

Firstly, we aimed to assess whether the elderly expert meditators showed differences in GM volume and FDG metabolism compared to elderly controls. For this purpose, w-score maps were computed for each expert meditator and each imaging modality using the 67 elderly controls aged 55–75 as the reference and regressing out the effects of age and education as detailed elsewhere^63^. For each modality, individual w-score maps were averaged across the elderly expert meditators to provide whole-brain profiles of GM volume and FDG metabolism changes compared to matched controls. One-sample t-tests were then performed on the GM volume and FDG metabolism w-scores maps and differences from 0 were assessed using a threshold of p (voxel-level uncorrected) <0.001 with a cluster-level FWE-corrected p < 0.05 threshold. These latter analyses, for GM volume and FDG metabolism respectively, were conducted to identify the clusters of interest.

Secondly, we wished to assess whether the brain regions exhibiting meditation-expertise-related effects were sensitive to aging and whether the elderly expert meditators differed from matched controls in this aging process. For this purpose, i) we performed voxelwise regression analyses between age and GM volume or FDG metabolism, including education as a covariate, within the entire group of 186 controls aged 20 to 87 years; ii) we extracted, in the 186 controls aged 20 to 87 years, the values of GM volume and FDG metabolism in the clusters of interest. The effect of education were regressed out from these extracted values and the education-adjusted values were plotted against age across the 186 controls. The values of the elderly expert meditators, similarly corrected for education, were reported on these plots.

#### Complementary analyses

Firstly, group comparison analyses were conducted on measures of cognition, lifestyle, and sleep quality, (missing data indicated in Supplementary Table S1), using both non-parametric Mann-Whitney U-test and ANCOVAs to correct for education. Differences were considered as marginal when p < 0.05 and as significant when p < 0.01.

Secondly, to investigate possible changes in hippocampus substructures, two different approaches were used. First, the raw volumetric measures of the subiculum, CA1 and CA2/3/4/DG obtained from manual delineation were normalized by the total intracranial volume (derived from the segmentation of the T1-MRI with SPM12) to compensate for inter-individual variability in head size, and compared between groups using both non-parametric Mann-Whitney U-test and ANCOVAs to correct for education. To limit the number of statistical tests, and as there was no significant effect of laterality (left versus right), the analyses were performed on the bilateral (left plus right) measurements. Secondly, 3D-hippocampal surface projection was also performed as described in details elsewhere^64^. Briefly, the SPM-T map of the one-sample t-test performed on the GM volume w-scores maps of the elderly expert meditators was superimposed onto a 3D template surface representation of the right and left hippocampi using the publicly available “Anatomist/BrainVISA” software (http://www.brainvisa.info/).

Thirdly, if significant differences were found between elderly expert meditators and elderly controls in their lifestyle measures, we tested whether the differences in the neuroimaging measures were still significant when correcting for these lifestyle measures. ANCOVAs were performed on the extracted GM volume or FDG metabolism values within the clusters of interest comparing both groups and including education and the lifestyle measures as covariates.

Finally, a subgroup of the elderly controls was composed by excluding all elderly with less than 12 years of education–the minimum in the elderly expert meditators–and/or with an Aβ-positive Florbetapir PET scan (n = 31; see demographic information in Table 1). Values of GM volume and FDG metabolism were extracted in the clusters of interest in the 6 elderly expert meditators and in the 31 Aβ-negative education group-matched elderly controls and Mann-Whitney U tests were used to compare both groups.

### Data Availability

The datasets generated and or analyzed during the current study are available from the corresponding author on reasonable request.

## Electronic supplementary material

Supplementary Information
