# Supplementary material for: Reduced age-associated brain changes in expert meditators: a multimodal neuroimaging pilot study
Source: Sci Rep. 2017 Aug 31;7:10160. doi: 10.1038/s41598-017-07764-x (PMC5578985; doi:10.1038/s41598-017-07764-x)
Supplement: Supplementary file 1 — Supplementary Information [file 41598_2017_7764_MOESM1_ESM.pdf]

Reduced age-associated brain changes in expert meditators: a multimodal neuroimaging pilot study

Gaël Chételat, Florence Mézenge, Clémence Tomadesso, Brigitte Landeau, Eider Arenaza-Urquijo, Géraldine Rauchs, Claire André, Robin de Flores, Stéphanie Egret, Julie Gonneaud, Géraldine Poisnel, Anne Chocat, Anne Quillard, Béatrice Desgranges, Jean-Gérard Bloch, Matthieu Ricard, Antoine Lutz

Supplementary Information

Supplementary Table S1. Neuropsychological, lifestyle, sleep and hippocampal volume data in the expert-meditators and elderly control samples.

	Elderly controls		Expert meditators	Mann-Whitney p-value	ANCOVA p-value
	n	Mean ± SD	Mean ± SD		
Neuropsychological scores					
Fluency	67	55.9 ± 14.3	62.5 ± 12.7	0.3	0.9
Memory	67	16.5 ± 4.2	20.3 ± 3.3	**0.02**	0.2
Span forward	66	6.0 ± 1.2	6.7 ± 1.6	0.4	0.6
Span backward	66	4.8 ± 1.2	5.2 ± 1.2	0.3	0.6
Processing speed (TMT A sec)	66	39.0 ± 11.7	34.3 ± 8.5	0.4	0.6
Executive functions (TMT B-A)	66	45.8 ± 19.2	40.0 ± 19.2	0.6	0.7
Lifestyle measures					
Mental activity (≤30 yrs)	43	19.0 ± 4.7	25.0 ± 4.5	**<u>0.008</u>**	0.05
Lifelong occupation	43	44.5 ± 13.7	57.3 ± 6.0	**0.01**	0.3
Mental activity (30-65 yrs)	43	19.0 ± 5.0	24.2 ± 2.9	**0.01**	0.2
Diet	43	6.0 ± 2.1	8.3 ± 2.1	**0.02**	**0.01**
Sleep measures					
Sleep quality	51	4.2 ± 1.1	5.0 ± 0.9	0.1	0.2
Sleep latency (min.)	51	19.1 ± 17.4	10.3 ± 7.9	0.2	0.3
Sleep duration (hours)	51	7.0 ± 1.1	6.9 ± 1.2	0.8	0.4
Number of awakenings	50	1.7 ± 1.2	1.0 ± 0.6	0.1	0.3
Duration of awakenings (min.)	51	34.7 ± 33.9	5.0 ± 3.2	**<u>0.009</u>**	**0.03**
Hippocampus volume					
Entire hippocampus	53	3.21 ± 0.31	3.44 ± 0.28	0.09	0.07
CA1	53	1.02 ± 0.16	1.16 ± 0.13	**0.04**	**0.03**
Subiculum	53	0.97 ± 0.12	1.06 ±0.09	0.08	0.1
CA2/3/4/DG	53	1.21 ± 0.15	1.22 ± 0.11	1.0	0.8

Between group differences were assessed using non-parametric Mann-Whitney U-tests and ANCOVAs controlling for education. P values < 0.05 (marginal effects) are in bold and p values < 0.01 are in bold and underlined.

Supplementary Table S2. Between-group differences (p-values) in grey matter volume and FDG metabolism in the clusters of interests (shown in Figures 1C and 2C) when comparing the 6 elderly expert meditators and the 67 elderly controls with ANCOVAs correcting for education and all lifestyle measures.

Clusters of interest	P value
MRI-cluster 1: VMPC-ACC	0.003
MRI-cluster 2: L TP junction	0.02
MRI-cluster 3: R TP junction	0.002
FDG-cluster 1: VMPC-ACC	0.0001
FDG-cluster 2: R Insula	0.001
FDG-cluster 3: PCC	0.007

VMPF: ventromedial prefrontal cortex; **ACC**: anterior cingulate cortex; **TP**: temporo-parietal; **PCC:** posterior cingulate cortex; **L:** left; **R:** right.

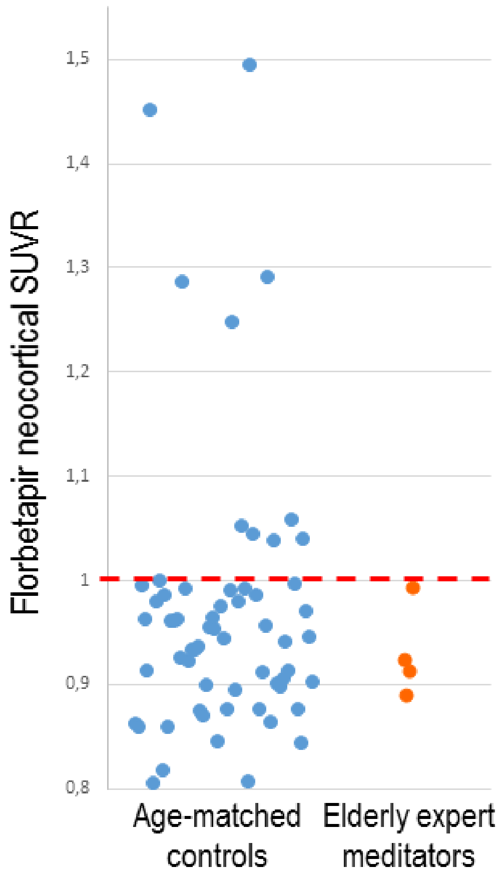

Supplementary Figure S1. Plot of the neocortical florbetapir SUVR values in the 51 controls from the elderly control group (blue) and the 4 expert meditators (orange) who had a florbetapir-PET scan. The threshold for Aβ-positivity was determined from Florbetapir-PET data acquired in a group of 41 healthy adults below 40 years old from the IMAP+ study [1,2].

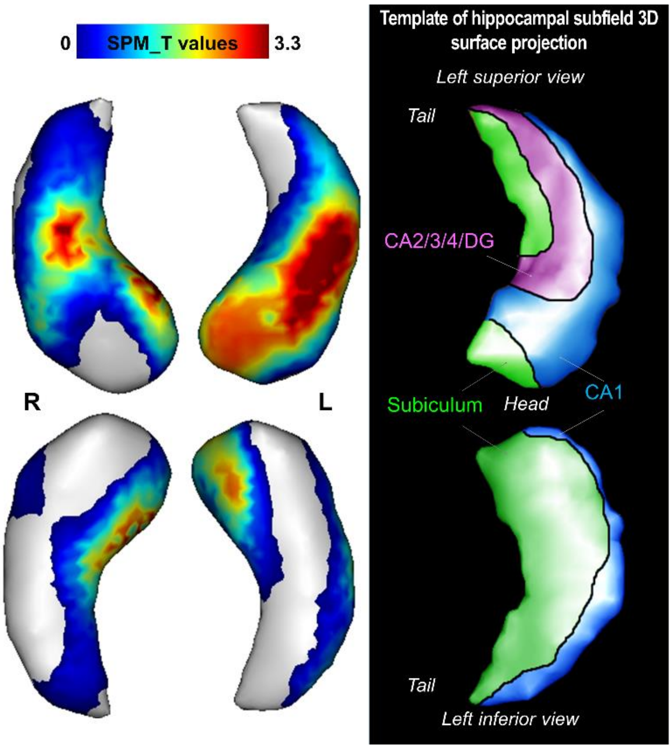

Supplementary Figure S2. **3D hippocampal surface representation of the one-sample analysis on elderly expert meditators GM volume w-scores maps.** Regions of highest SPM-T values, i.e. where meditators volume w-scores are the most higher than zero when referring to the elderly control group, are indicated in red and mostly corresponds to the left CA1 subfield. A template of the hippocampal subfield topography on the left superior and inferior views of the 3D hippocampal surface is provided for reference (see[3]).

References

1. Besson, F. L. *et al.* Cognitive and Brain Profiles Associated with Current Neuroimaging Biomarkers of Preclinical Alzheimer's Disease. *J. Neurosci. Off. J. Soc. Neurosci.* **35,** 10402–10411 (2015).
2. La Joie, R. *et al.* Qualitative and quantitative assessment of self-reported cognitive difficulties in nondemented elders: Association with medical help seeking, cognitive deficits, and β-amyloid imaging. *Alzheimers Dement. Amst. Neth.* **5,** 23–34 (2016).
3. Chételat, G. *et al.* Three-dimensional surface mapping of hippocampal atrophy progression from MCI to AD and over normal aging as assessed using voxel-based morphometry. *Neuropsychologia* **46,** 1721–1731 (2008).
